# Toll like receptor 2 is overexpressed in FMF patients during attacks and inhibited by colchicine treatment

**DOI:** 10.1186/1546-0096-13-S1-P74

**Published:** 2015-09-28

**Authors:** H Ben-David, V Hornung, T Ebert, A Livneh, I Ben-Zvi

**Affiliations:** 1 Sheba Medical Centre tel-Hashomer, Heller Institute of Medical Research, Ramat-Gan, Israel; 2 Bonn University, Institute for Molecular Medicine, Bonn, Germany; 3 Sheba Medical Centre Tel-Hashomer, Rheumatology unit & Department of Internal Medicine F, Ramat Gan, Israel; 4 Tel-Aviv University, Sackler Faculty of Medicine, Tel-Aviv, Israel

## Background

FMF is a systemic auto-inflammatory disorder, characterized by recurrent episodes of fever and serosal inflammation. The *MEF* gene, which is associated with FMF, encodes for the protein pyrin. FMF associated mutations, interrupt with pyrin normal function, leading to activation of the innate immune system and overexpression of IL-1β, and consequently to a systemic inflammatory response. Toll-like receptors (TLRs) play an essential role in the innate immune responses, by recognition of pathogen-associated molecular patterns and endogenous peptides. TLRs trigger a cascade of signaling events, leading to cytokine production. TLR2 is implicated in several inflammatory conditions, but its role in the pathogenesis of FMF is not completely clear.

## Objectives

To study the role of TLR2 in the inflammatory process of FMF.

## Materials and methods

We tested TLR2 naïve expression on monocytes of FMF attack-free patients (n=20) by FACS. We further tested the effect of sera from FMF patients in acute attack (n=6) on TLR2 expression by monocytes of healthy controls. The role of TLR2 was studied in respect to *MEFV* mutation, performed in THP-1 cells. TLR2 downstream signaling was studied by ELISA to measure IL-1β secretion, or by Western-blot to measure NF-κB.

## Results

FMF attack-free patients have increased CD14^+^TLR2^+^ cell-count, as compared to healthy donors. High dose of colchicine treatment (≥2mg/d) inhibited this increased expression of TLR2 in FMF patients. Colchicine *in vitro* also inhibited the levels of TLR2 expression on THP-1 cells. Sera from FMF patients in acute attack induced TLR2 expression by both monocytes of healthy donors and THP-1 cells, and IL-1β secretion in healthy monocytes, and colchicine inhibited this induction. Furthermore, TLR2 agonist (Pam2CSK4) increased the secretion of IL-1β by PBMCs of healthy donors, and this activation was inhibited by colchicine. In *MEFV* -mutated THP-1 cells, TLR2 expression was spontaneously up-regulated by 3.8 folds, while TLR4 expression was elevated by 2 folds as compared to wild-type. Wild-type THP-1 cells presented elevated NF-κB expression when cultured with Pam2CSK4, whereas colchicine treatment abolished this expression. *MEFV*-mutated THP-1 cells expressed elevated levels of NF-κB, as compared to their wild-type counterparts.

## Conclusion

TLR2 activation is up-regulated in monocytes of FMF patients, and colchicine inhibits this up-regulation *in-vitro* and *in-vivo*. Elevated expression of TLR2 promotes IL-1β production, and thus contribute to the uncontrolled inflammation manifested in FMF.

